# The Marker of Tubular Injury, Kidney Injury Molecule-1 (KIM-1), in Acute Kidney Injury Complicating Acute Pancreatitis: A Preliminary Study

**DOI:** 10.3390/jcm9051463

**Published:** 2020-05-13

**Authors:** Justyna Wajda, Paulina Dumnicka, Witold Kolber, Mateusz Sporek, Barbara Maziarz, Piotr Ceranowicz, Marek Kuźniewski, Beata Kuśnierz-Cabala

**Affiliations:** 1 Jagiellonian University Medical College, Faculty of Medicine, Department of Anatomy, 31-034 Kraków, Poland; justynawajda87@tlen.pl (J.W.); msporek1983@gmail.com (M.S.); 2 Jagiellonian University Medical College, Faculty of Pharmacy, Department of Medical Diagnostics, 30-688 Kraków, Poland; paulina.dumnicka@uj.edu.pl; 3 Department of Surgery, Complex of Health Care Centers in Wadowice, 34-100 Wadowice, Poland; w.kolber@wp.pl; 4 Jagiellonian University Medical College, Faculty of Medicine, Chair of Clinical Biochemistry, Department of Diagnostics, 31-501 Kraków, Poland; mbmaziar@cyf-kr.edu.pl; 5 Jagiellonian University Medical College, Faculty of Medicine, Department of Physiology, 31-531 Kraków, Poland; 6 Jagiellonian University Medical College, Faculty of Medicine, Department of Nephrology, 30-688 Kraków, Poland; marek.kuzniewski@uj.edu.pl

**Keywords:** kidney injury molecule-1, acute pancreatitis, acute kidney injury, biomarkers of acute kidney injury

## Abstract

Acute pancreatitis (AP) may be associated with severe inflammation and hypovolemia leading to organ complications including acute kidney injury (AKI). According to current guidelines, AKI diagnosis is based on dynamic increase in serum creatinine, however, creatinine increase may be influenced by nonrenal factor and appears late following kidney injury. Kidney injury molecule-1 (KIM-1) is a promising marker of renal tubular injury and it has not been studied in AP. Our aim was to assess if urinary KIM-1 may be used to diagnose AKI complicating the early stage of AP. We recruited 69 patients with mild to severe AP admitted to a secondary care hospital during the first 24 h from initial symptoms of AP. KIM-1 was measured in urine samples collected on the day of admission and two subsequent days of hospital stay. AKI was diagnosed based on creatinine increase according to Kidney Disease: Improving Global Outcomes 2012 guidelines. Urinary KIM-1 on study days 1 to 3 was not significantly higher in 10 patients who developed AKI as compared to those without AKI and did not correlate with serum creatinine or urea. On days 2 and 3, urinary KIM-1 correlated positively with urinary liver-type fatty acid-binding protein, another marker of tubular injury. On days 2 and 3, urinary KIM-1 was higher among patients with systemic inflammatory response syndrome, and several correlations between KIM-1 and inflammatory markers (procalcitonin, urokinase-type plasminogen activator receptor, C-reactive protein) were observed on days 1 to 3. With a limited number of patients, our study cannot exclude the diagnostic utility of KIM-1 in AP, however, our results do not support it. We hypothesize that the increase of KIM-1 in AKI complicating AP lasts a short time, and it may only be observed with more frequent monitoring of the marker. Moreover, urinary KIM-1 concentrations in AP are associated with inflammation severity.

## 1. Introduction

Acute pancreatitis (AP) is a common acute gastrointestinal inflammatory condition requiring hospital treatment. In 15%–20% of patients, the severity of inflammatory response may lead to organ injury and transient or persistent organ failure. Severe acute pancreatitis (SAP) is diagnosed in patients with organ failure lasting over 48 h, and is associated with substantial mortality [1,2].

AP is initiated by uncontrolled activation of pancreatic enzymes, including trypsin. The injury to pancreatic cells (autodigestion) induces inflammation. Moreover, early events in AP involve nuclear factor κB activation in pancreatic acinar cells, with resulting cytokines’ production [3]. Local inflammation develops, self-limited in mild cases, and followed by systemic inflammation in severe AP. Neutrophil recruitment, cytokine storm, oxidative stress, endothelial dysfunction, and injury leading to increased vascular permeability and hemodynamic complications in severe cases are the features of systemic inflammation that may lead to organ dysfunction including lungs, cardiovascular system, and kidneys [1,2]. Acute kidney injury (AKI) is among the most common complications of SAP [4,5]. It may develop in early stages as a result of dehydration, increased vascular permeability, fluid redistribution, and acute inflammation, however, it may also be a late complication related to sepsis [4].

AKI is defined by Kidney Disease Improving Global Outcomes (KDIGO) criteria as a ≥50% increase in plasma creatinine concentration over baseline within 7 days or an increase in serum creatinine by 0.3 mg/dL within 2 days or a decreased (<0.5 mL/kg/h) urine output lasting at least 6 h [6]. In the course of AP, AKI is associated with adverse prognosis and mortality up to 40% [4,5], however, much better outcomes have been reported when AKI was an isolated complication of SAP [7]. AKI may also result in chronic kidney disease, moreover, acute-on-chronic kidney injury is associated with a high risk of end-stage renal disease [8,9]. Although currently the diagnosis of AKI is based on serum creatinine increase, creatinine is recognized as a late marker of AKI, significantly increasing after 24–36 h from kidney injury [10,11]. Serum creatinine increase is not specific to kidney injury, rather, it is a marker of decreasing kidney function. Therefore, there is a need for laboratory markers that enable early detection of renal injury.

Kidney injury molecule-1 (KIM-1) is a promising biomarker of kidney injury. Its expression in proximal renal tubular epithelial cells (mainly S3 segment) is highly elevated at the early stage of AKI [12,13] and increasing urinary KIM-1 levels are associated with more advanced renal injury [8]. KIM-1 is a transmembrane glycoprotein with molecular mass of 104 kDa, a member of the transmembrane immunoglobulin and mucin domain (TIM) family of proteins and immunoglobulin superfamily [13,14]. Normal kidney tissues express trace KIM-1, while increased KIM-1 expression has been observed in AKI resulting from ischemia, hypoxia, or toxicity [8]. Moreover, increased expression was reported in tubulointerstitial nephropathies and polycystic kidney disease [8,15]. The ectodomain of KIM-1 (90 kDa) is cleaved by matrix metalloproteinases and released into the urine, and this mechanism is also upregulated in proximal tubule injury [16]. In a meta-analysis of 11 studies including almost 3000 patients, Shao et al. [14] estimated diagnostic sensitivity and specificity of KIM-1 in AKI for 74% and 86%, respectively. However, they also reported that urine KIM-1 concentrations may be significantly influenced by comorbidities, including diabetes, hypertension, and atherosclerosis [14].

KIM-1 seems to have several advantages over other markers of kidney injury. It was shown to increase in urine shortly after injury, before renal tubular damage could be observed in histological examination [17]. Huang et al. [18] confirmed that KIM-1 was increased within 24 h after kidney injury. Han et al. [19] reported that urinary KIM-1 can differentiate ischemic AKI from prerenal azotemia and chronic kidney disease, and its urinary concentrations were not influenced by urinary tract infections.

To our best knowledge, KIM-1 has not been evaluated in AKI complicating acute pancreatitis. The aim of this preliminary study was to verify if KIM-1 may be a useful laboratory marker in the clinical setting of AKI developing in the early phase of AP.

## 2. Experimental Section

### 2.1. Patients

The retrospective study included 69 patients (51 men, 18 women) with the diagnosis of AP admitted to the Department of Surgery, Complex of Health Care Centers in Wadowice, Poland (a secondary care hospital), between March 2014 and December 2015. The diagnosis of AP was based on revised 2012 Atlanta classification, that is, AP was diagnosed when at least two of the following criteria were met: abdominal pain suggestive of AP; AP signs in abdominal imaging (magnetic resonance imaging, contrast-enhanced computed tomography, or ultrasonography); serum amylase or lipase above three times the upper reference limit [20]. Patients were recruited within 24 h from hospital admission according to the study protocol. The study included patients with symptoms of AP lasting shorter than 24 h before hospital admission. Patients with chronic pancreatitis, active cancer, or chronic liver diseases (viral hepatitis, liver cirrhosis) were excluded from the study. Only the patients who provided written informed consent were included.

The study protocol included collection of demographic and clinical data described below, and collection of blood and urine samples during the first three days of hospital stay. Our primary interest in this study was whether urinary KIM-1 measured in samples collected on days 1 and 2 from admission enables the prognosis or diagnosis of AKI developing in the early phase (the first week) of AP. In a part of patients, KIM-1 was also measured in urine collected on day 3, to study dynamic changes in the urinary concentrations.

The study protocol was approved by the Bioethics Committee of the Beskidy Medical Chamber, Bielsko-Biała, Poland (approval number 2014/02/06/1 issued on 6 February 2014).

The demographic and clinical data were collected on recruitment and during the hospital stay of the patients. These included information on age and sex, comorbidities (cardiovascular disease, diabetes, renal disease, body mass index >30 kg/m^2^, i.e., obesity), AP etiology, imaging findings of pancreatic necrosis, systemic inflammatory response syndrome (SIRS) during the first three days of hospital stay, organ failure, intensive care unit (ICU) treatment, use of surgery or parenteral nutrition during the hospital stay, length of hospital stay, severity of AP defined according to 2012 Atlanta classification [2], and outcome.

The clinical and laboratory values on the first day of hospital stay were used to calculate the bedside index of severity in AP (BISAP) [21]. The data obtained during the first 48 h of hospital stay were used to calculate the Ranson’s score [22]. AKI was defined using criteria of Kidney Disease Improving Global Outcomes (KDIGO) based on increase in serum creatinine (>50% over a week or 26.5 µmol/L over 48 h) [23]. SIRS has been diagnosed in line with the definition cited in 2012 Atlanta classification [2] as the presence of two or more of the following criteria: heart rate >90 beats/min; core temperature <36 °C or >38 °C; white blood count <4000 or >12000/µL; (4) respirations >20/min or PCO_2_ < 32 mm Hg.

### 2.2. Laboratory Tests

Blood and urine samples were collected on admission (study day 1) and two following days (study days 2 and 3; there were no deaths during the first three days of observation). Routine laboratory tests were done on the day of collection, that is, complete blood counts with leukocyte differential, biochemistry (serum amylase, lactate dehydrogenase, albumin, total calcium, bilirubin, glucose, urea, creatinine, C-reactive protein—CRP) and citrated plasma D-dimer, using automated analyzers: Sysmex XN hematology analyzer (Sysmex Corporation, Cobe, Japan), Cobas E411 (Roche Diagnostics, Mannheim, Germany) and Vitros 5600 (Ortho Clinical Diagnostics, Raritan, NJ, USA) biochemistry and immunochemistry analyzers, and Coag XL (Diagon, Budapest, Hungary) coagulation analyzer. Excess serum and urine samples collected on study days 1–3 were aliquoted and stored at −80 °C, and were further used to assess serum concentrations of soluble fms-like tyrosine kinase-1 (sFlt-1), procalcitonin and urokinase-type plasminogen activator receptor (uPAR), and urine concentrations of KIM-1 and liver-type fatty acid-binding protein (L-FABP).

The concentrations of sFlt-1 and procalcitonin were measured by electrochemiluminescence on Cobas 8000 analyzer (Roche Diagnostics, Mannheim, Germany) in the Diagnostic Department of University Hospital, Krakow, Poland. Serum concentrations of uPAR were measured using Quantikine ELISA Human uPAR Immunoassay (R & D Systems, McKinley Place, MN, USA). The minimum detectable dose for uPAR was <33 pg/mL; the mean serum concentration in healthy volunteers was 2370 pg/mL (range 1195–4415 pg/mL) according to the manufacturer of the test.

Urinary KIM-1 and L-FABP concentrations were measured by enzyme immunoassays using commercially available kits. Urinary L-FABP was measured in samples that remained after KIM-1 measurements. Urinary KIM-1 was assessed with Quantikine ELISA Human TIM-1/KIM-1/HAVCR Immunoassay (R & D Systems, McKinley Place, MN, USA). Patients’ samples were tested in series, in duplicates, according to the manufacturer’s instructions. Minimum detectable dose for KIM-1 concentration in urine was 0.009 ng/mL; the normal range determined by the manufacturer of the kit was between 0.156 and 5.33 ng/mL. Urinary L-FABP concentrations were measured with Human L-FABP Assay (CMIC Holding Co., Tokyo, Japan). The sensitivity of the assay was 3 pg/mL. The readings were made with an automatic microplate reader, Automatic Micro ELISA Reader ELX 808 (BIO-TEK Instruments Inc., Winooski, VT, USA). The measurements were performed in the Department of Diagnostics, Chair of Clinical Biochemistry, Jagiellonian University Medical College, Krakow, Poland.

### 2.3. Statistical Analysis

Categorical data were reported as number of patients (*n*) and percentage of the appropriate group. Pearson’s chi-squared test was used to compare categorical data between groups. Mean ± standard deviation (SD) or median (lower; upper quartiles) were reported for normally or non-normally distributed quantitative variables, respectively. Distributions were assessed for normality using the Shapiro–Wilk test. The differences between groups were assessed with t-test or Mann–Whitney’s test. Spearman’s rank coefficient was computed for simple correlations of urinary KIM-1 as the variable was non-normally distributed. The Friedman’s test was used to analyze changes over time in urinary concentrations of KIM-1 and L-FABP. All statistical tests were two-tailed and the *p*-values of <0.05 indicated significant results. The calculations were made with the use of Statistica 12 software (StatSoft, Tulsa, OK, USA).

## 3. Results

In the studied group of 69 patients, there were 21 (30%) patients with mild AP, 44 (64%) with moderately severe, and 4 (6%) with severe AP. Ten patients (14%) developed AKI in the early phase of AP, diagnosed according to KDIGO criteria. We observed no significant differences in the etiology of AP and baseline comorbidities between patients with and without AKI (Table 1). However, the proportion of patients with moderately severe and severe AP tended to be higher among patients with AKI (90% versus 66%), which was accompanied by more prevalent ICU treatment and higher mortality (Table 1). All patients with AKI were men, a significant difference in comparison with non-AKI patients (Table 1).

As expected, serum creatinine and urea were significantly higher in the AKI group compared to non-AKI subjects. Moreover, significantly higher concentrations of serum uPAR and procalcitonin, and significantly higher activities of lactate dehydrogenase were observed in patients with AKI on the day of hospital admission (Table 2).

**Figure 1:**
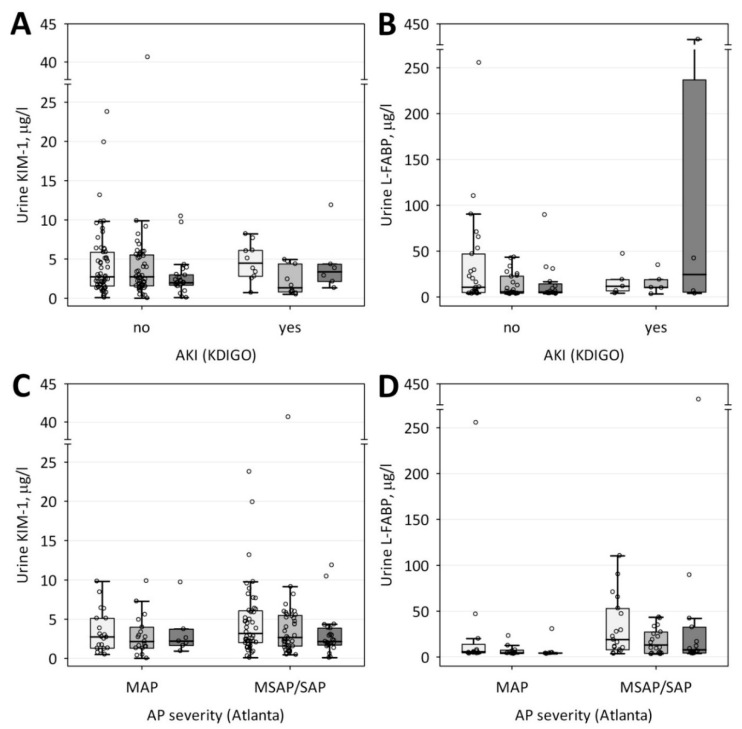
Urinary concentrations of the studied markers of tubular injury: KIM-1 (**A**,**C**) and L-FABP (**B**,**D**) among patients with and without acute kidney injury (AKI) (**A**,**B**) and with mild acute pancreatitis (MAP) in comparison with moderately severe (MSAP) and severe (SAP) disease (**C**,**D**). Data are shown as median (central line), lower/upper quartile (box), and nonoutlier range (whiskers), circles denote the row data. Light gray boxes denote the concentrations measured on day 1, medium grey on day 2, and dark grey on day 3.

Neither KIM-1 nor L-FABP urinary concentrations differed significantly between patients with and without AKI on the day of admission (Table 2). Consequently, the studied markers on days 2 and 3 of the study did not differ between the groups (Figure 1A,B). Most remarkably, no correlations were observed between KIM-1 and serum urea or creatinine during the study (Table 3). Also, L-FABP did not correlate with urea or creatinine.

Men and women did not differ in KIM-1 and L-FABP concentrations. We did not observe significant changes in urinary KIM-1 or L-FABP over the three days of the study.

No significant differences in the concentrations of both urinary markers of tubular injury were observed between patients with mild and more severe AP throughout the study (Figure 1C,D), although day 1 (*p* = 0.06) and day 2 (*p* = 0.07) concentrations of L-FABP tended to be higher in patients with moderately severe AP and SAP as compared with mild AP. Neither KIM-1 nor L-FABP correlated significantly with prognostic scores (BISAP and Ranson’s), and only day 1 concentrations of KIM-1 positively correlated with the length of hospital stay (R = 0.35; *p* = 0.004).

**Figure 2:**
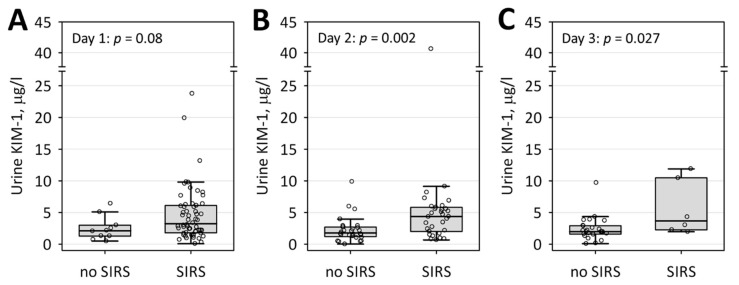
Urinary concentrations of KIM-1 among patients with and without systemic inflammatory response syndrome (SIRS) on day 1 (**A**), 2 (**B**), and 3 (**C**) of the study. Data are shown as median (central line), lower/upper quartile (box), and nonoutlier range (whiskers), circles denote the row data.

Statistically significant positive correlations were noted between urinary KIM-1 and the inflammatory markers: serum CRP, uPAR, procalcitonin and blood neutrophil count on day 1 of the study, CRP and D-dimer on day 2, and procalcitonin on day 3 (Table 3). Also, KIM-1 negatively correlated with serum albumin on day 3 of the study. Moreover, higher concentrations of KIM-1 in urine were observed among patients with SIRS on day 2 and 3 of the study (Figure 2). Additionally, we observed positive correlations between KIM-1 and lactate dehydrogenase on days 2 and 3, and negative correlation with hematocrit on day 3.

Similar correlations were observed in the case of urinary L-FABP, although because of low number of available samples, we were only able to confirm the strongest associations. On day 1, L-FABP correlated positively with hematocrit (R = 0.41; *p =* 0.023), on days 2 and 3 with lactate dehydrogenase (R = 0.58, *p =* 0.005; and R = 0.79, *p* < 0.001, respectively), CRP (R = 0.61, *p =* 0.001 and R = 0.60, *p =* 0.006), and with D-dimer (R = 0.47, *p =* 0.015 and R = 0.72, *p* < 0.001). Moreover, on day 3, we observed positive correlation between L-FABP and procalcitonin (R = 0.63; *p =* 0.005). Both markers (KIM-1 and L-FABP) were significantly correlated on days 2 and 3 of the study (Table 3).

## 4. Discussion

Although diagnostic usefulness of urinary KIM-1 has been studied in various clinical settings (including AKI in intensive care patients, surgical patients, including cardiovascular surgery, in obstructive nephropathy, and cisplatin-induced kidney injury) [13,14,24,25,26,27,28], there are no data addressing its diagnostic utility in prediction or diagnosis of AKI complicating AP. Our preliminary study assessed the concentrations of KIM-1 in urine of patients in the first 72 h of AP of various severities, in order to obtain data on the possibilities of using the marker for early prognosis of AKI complicating AP.

In the studied group, we were not able to show statistically significant differences in urinary KIM-1, neither between patients who developed AKI in the early stage of AP in comparison to those who did not, nor between those with moderately severe to severe AP in comparison to mild AP. Urinary KIM-1 did not correlate with markers of kidney function, namely, serum creatinine and urea, however, on days 2 and 3 of the study, it correlated with another marker of tubular injury, that is, L-FABP. To the contrary, significant positive correlations were observed between urinary KIM-1 and the markers of inflammation: serum CRP, uPAR, procalcitonin and blood neutrophil count on day 1, CRP and D-dimer on day 2, and procalcitonin on day 3, and negative correlation with serum albumin on day 3 of the study. Consequently, higher urinary KIM-1 concentrations have been observed in patients with SIRS. Moreover, we observed positive correlation between day 1 urinary KIM-1 and the length of hospital stay.

KIM-1 has been associated with inflammation in renal tubular injury. Although the role of KIM-1 in AKI has been usually viewed as anti-inflammatory (as a receptor to phosphatidylserine, it increases the uptake of apoptotic and necrotic bodies) and involved in tubular cells’ repair [15], Tian et al. [26] showed that KIM-1 plays an important role in macrophage migration to injured tubular cells in AKI, and the mitogen-activated protein kinase signaling pathway may be involved in this process. van Timmeren et al. [29] reported that in various kidney diseases, KIM-1 expression in kidney bioptates was detected in areas of inflammation and fibrosis, while urinary KIM-1 increased in parallel to increased tissue expression and correlated with inflammation.

In the studied group, uPAR and procalcitonin concentrations were higher in patients with AKI. In AP, inflammation plays a significant role in pathophysiology of AKI [4]. Tang et al. [30] discuss the role of CRP in AKI, pointing to its role in pathogenesis of AKI. CRP is involved in development of AKI and the increased serum concentrations of CRP correlate with AKI severity. The in vitro study by Castellani et al. [31] has shown that CRP activates the mitogen-activated protein kinase pathway and upregulates regulated on activation, normal T cell expressed and secreted (RANTES) expression, which is expressed and secreted by T cells and plays a key role in recruiting leukocytes into inflammatory sites. Bear et al. [32] confirmed the effect in human renal distal tubular cells. Li et al. [33] reported increased activation of both nuclear factor κB/p65 and transforming growth factor-β/Smad3 signaling being the major mechanism involved in the process of CRP-promoting AKI at acute setting. Procalcitonin is also a marker of inflammation, not only in acute bacterial sepsis, but in other inflammatory disorders, and has been associated with AKI development in sepsis [34]. Moreover, elevated procalcitonin has been previously observed in AKI complicating AP, similarly to present results [35].

In our study, both urinary KIM-1 and L-FABP correlated positively with serum lactate dehydrogenase starting from day 2 of the study. Moreover, patients with AKI presented with higher LDH activities already on admission. LDH may be viewed as a marker of tissue ischemia and necrosis. In AP, hypovolemia and ischemia contribute to renal tubular injury [4,5,36]. The duration of renal ischemia has been well recognized in clinical studies to be associated with the severity and progression of AKI [37]. Increased urinary KIM-1 has been observed in a rat model of ischemia-reperfusion AKI [38]. In mice, KIM-1 increased in serum and urine during the first 3 h after kidney injury and increased serum KIM-1 has also been recently reported in patients with AKI [39]. Also, increased urinary L-FABP, a 14-kDa fatty acid-binding protein elevated and secreted into the urine as a result of reactive oxygen stress due to renal ischemia, has been shown to correlate with insufficient renal peritubular capillary blood flow and the progression of AKI [40]. Nonetheless, we were not able to show increased urinary KIM-1 or L-FABP in early-stage AP patients with AKI.

One explanation may be that the time frame of urine sampling in our patients may be inappropriate. There is evidence that urinary KIM-1 increase in response to kidney injury lasts a short time. In patients who underwent cardiopulmonary bypass surgery, the dynamics of KIM-1 increase has been analyzed by Shao et al. [14]. The diagnostic sensitivity of urinary KIM-1 was highly dependent on time following the surgery, with a maximum value of 90% between 2 and 6 h after the insult, and decreasing values thereafter (74% at 12 h after surgery) [14]. In our study, single measurements were performed on each of the three days of the study. The time from initial symptoms of AP to admission was not uniform in our patients, as was the time of serum creatinine increase. Considering the results of Shao et al., it may be possible that more measurements are needed to detect the increase in urinary KIM-1 in patients developing AKI in the course of AP. Although KIM-1 has been described over 20 years ago, and first commercially available tests have been introduced in 2002, the automated and robust methods of measurement of KIM-1 concentrations in urine are still not available, making it difficult to use the marker in clinical settings. Considering the lack of automated assays and the difficulties in assessing the optimum time frame of KIM-1 measurements, it is not feasible now to use KIM-1 to diagnose AKI in clinical practice.

Based on our previous studies and literature search, we must say that none of the biomarkers that have been studied in this setting are good enough to efficiently and early predict AKI complicating the course of AP [5,41]. Most promising results have been obtained for serum cystatin C, a functional marker of glomerular filtration [42] and for urinary NGAL, a marker of tubular injury [43]. Of note, serum cystatin C measurements have been automated and the standardization of measurements has been available since 2010 [44]. Also, urinary NGAL may be measured with automated methods, and although the results obtained by various methods are not directly comparable, the turn-around times are 2–3 h, enabling the use of this marker for fast diagnosis. Both serum cystatin C and urinary NGAL seem to rise quickly in AP complicated by AKI and be useful in early prognosis (in first 24 h) [42,43,45,46]. In clinical practice, however, serum creatinine remains the marker of choice to diagnose AKI, although clinicians are aware that its concentrations depend on muscle mass, changing with race, sex, and nutritional status, and are influenced by fluid abnormalities or liver insufficiency in critical illness. Therefore, in systemic inflammatory state associated with AP, it may be difficult to diagnose AKI early on the basis of serum creatinine changes [10,47]. Serum creatinine depends on the volume of distribution, thus, the fluid therapy in AP may significantly dilute creatinine concentrations, delaying the diagnosis of AKI [10]. The biomarkers of tubular injury may potentially enable earlier diagnosis or prognosis of AKI; moreover, they may enable more specific diagnosis, informing about the site of injury and possibly the mechanism of injury. However, more studies are needed to obtain robust data on their diagnostic accuracy before they can be introduced to clinical practice.

Our study has several limitations. As it was meant as a pilot study, its major limitation is a low number of patients included and low number of patients with AKI. We might have missed the most severe cases of AP (and AKI) as the study was performed in a secondary care center. The design was retrospective, but the authors performing KIM-1 measurements were blind to the diagnosis of AKI and the severity of AP at the time of measurements. Moreover, we measured KIM-1 in urine samples that were stored frozen for several months, however, de Vrie [48] and Schuh et al. [49] have shown stability of urinary KIM-1 upon long-term storage at –80 °C. Urinary KIM-1 concentrations were not corrected for urinary creatinine concentrations, but noncorrected urinary concentrations have been reported in most studies on the use of KIM-1 for the prognosis or diagnosis of AKI in various clinical settings [14,24,25,28,50,51,52].

Based on the study, we cannot definitely exclude the diagnostic utility of KIM-1 in AP, however, our results do not support it. We may hypothesize that the increase of KIM-1 in AKI complicating AP lasts a short time, and it may only be observed with frequent monitoring of the marker. In summary, KIM-1 concentrations are correlated with severity of the inflammatory process in AP and do not seem to be a sensitive marker of AKI among patients in the early phase of AP.

## Figures and Tables

**Table 1:** Clinical characteristics of studied patients with acute pancreatitis.

Characteristic	AKI (*n* = 10)	No AKI (*n* = 59)	*p*-Value
Mean age ± SD, years	55.0 ± 16.9	46.9 ± 16.0	0.1
Male sex, *n* (%)	10 (100)	41 (69)	0.042
Comorbidities:			
any, *n* (%)	6 (60)	22 (37)	0.2
cardiovascular disease, *n* (%)	4 (40)	15 (25)	0.3
diabetes, *n* (%)	0	4 (7)	0.4
AP etiology:			
biliary, *n* (%)	4 (40)	15 (25)	0.7
alcohol, *n* (%)	2 (20)	20 (34)	
hyperlipidemia, *n* (%)	0	4 (7)	
idiopathic, *n* (%)	4 (40)	18 (31)	
other, *n* (%)	0	2 (3)	
Ranson’s score at first 48 h >3 points, *n* (%)	4 (40)	15 (25)	0.3
BISAP on day 1 ≥3 points, *n* (%)	4 (40)	13 (22)	0.2
Systemic inflammatory response syndrome on day 1, *n* (%)	9 (90)	49 (83)	0.6
Necrotizing AP, *n* (%)	1 (10)	7 (12)	0.9
AP severity:			
mild, *n* (%)	1 (10)	20 (34)	0.056
moderately severe, *n* (%)	7 (70)	37 (63)	
severe, *n* (%)	2 (20)	2 (3)	
Treatment in ICU, *n* (%)	2 (20)	2 (3)	0.037
Surgery, *n* (%)	1 (10)	3 (5)	0.5
Median length of hospital stay (lower; upper quartile), days	12 (10; 15)	12 (9; 15)	0.6
Mortality, *n* (%)	2 (20)	1 (2)	0.009

AKI, acute kidney injury; AP, acute pancreatitis; BISAP, bedside index of severity in acute pancreatitis; ICU, intensive care unit; SD, standard deviation.

**Table 2:** The results of laboratory test performed in the studied patients with acute pancreatitis on the day of admission. Data are shown as median (lower; upper quartile).

Laboratory Test	AKI (*n* = 10)	No AKI (*n* = 59)	*p*-Value
Amylase, U/L	443 (84; 677)	546 (172; 1812)	0.4
Lactate dehydrogenase, U/L	854 (661; 1027)	553 (472; 744)	0.010
Albumin, g/L	35 (31; 35)	36 (33; 40)	0.5
Total calcium, mmol/L	2.04 ± 0.21	2.16 ± 0.19	0.1
Bilirubin, µmol/L	39.2 (19.1; 68.7)	24.8 (13.7; 36.9)	0.2
Glucose, mmol/L	7.22 (6.78; 10.72)	7.33 (5.94; 8.78)	0.3
Creatinine, µmol/L	98.1 (91.9; 113.2)	68.1 (59.7; 74.3)	<0.001
Urea, mmol/L	7.50 (6.08; 9.00)	4.08 (3.17; 5.33)	0.002
Urine KIM-1, µg/L	4.47 (2.80; 6.10)	2.73 (1.57; 5.88)	0.2
Urine L-FABP, µg/L *	11.7 (6.8; 18.9)	10.5 (4.84; 46.83)	0.8
Hematocrit, %	46.3 (38.1; 48.7)	43.7 (40.7; 46.1)	0.3
White blood cells, ×10^3^/µL	14.1 (9.6; 19.2)	13.6 (11.4; 15.7)	0.9
Neutrophils, ×10^3^/µL	12.6 (11.2; 17.5)	11.0 (7.4; 14.7)	0.5
CRP, mg/L	122.0 (12.2; 253.4)	23.8 (8.70; 89.0)	0.3
uPAR, µg/L	5.34 (3.46; 6.28)	3.71 (2.82; 4.82)	0.042
Procalcitonin, µg/L	0.38 (0.23; 1.68)	0.14 (0.05; 0.36)	0.014
D-dimer, mg/L	1.80 (1.00; 6.06)	1.75 (0.87; 2.88)	0.5
sFlt-1, ng/mL	160 (108; 204)	136 (120; 161)	0.6

* The concentrations of L-FABP were only available in 5 patients with AKI and 26 patients without AKI. ALT, alanine aminotransferase; AST, aspartate aminotransferase; AKI, acute kidney injury; CRP, C-reactive protein; KIM-1, kidney injury molecule-1; L-FABP, liver-type fatty acid-binding protein; sFlt-1, soluble fms-like tyrosine kinase-1; uPAR, urokinase-type plasminogen activator receptor.

**Table 3:** Correlations between KIM-1 and the selected laboratory results on days 1, 2, and 3 of the study in the studied patients with acute pancreatitis.

Variable	Day 1 (*n* = 69)	Day 2 (*n* = 69)	Day 3 (*n* = 30)
Creatinine	R = 0.13; *p* = 0.3	R = − 0.20; *p* = 0.1	R = − 0.18; *p* = 0.4
Urea	R = 0.23; *p* = 0.07	R = 0.07; *p* = 0.6	R = 0.21; *p* = 0.3
Urine L-FABP	R = 0.12; *p* = 0.5	R = 0.54; *p* = 0.002 *	R =0.49; *p* = 0.028 *
Lactate dehydrogenase	R = 0.20; *p* = 0.1	R = 0.32; *p* = 0.029 *	R = 0.54; *p* = 0.005 *
Albumin	R = − 0.20; *p* = 0.2	R = − 0.22; *p* = 0.2	R = − 0.57; *p* = 0.020 *
Hematocrit	R = 0.08; *p* = 0.5	R = − 0.06; *p* = 0.7	R = − 0.38; *p* = 0.040 *
Neutrophils	R = 0.40; *p* = 0.021 *	R = 0.04; *p* = 0.8	R = 0.18; *p* = 0.4
CRP	R = 0.36; *p* = 0.004 *	R = 0.27; *p* = 0.045 *	R = 0.33; *p* = 0.08
uPAR	R = 0.30; *p* = 0.020 *	R = 0.20; *p* = 0.2	R = 0.29; *p* = 0.1
Procalcitonin	R = 0.32; *p* = 0.012 *	R = 0.13; *p* = 0.3	R = 0.39; *p* = 0.042 *
D-dimer	R = 0.06; *p* = 0.6	R = 0.31; *p* = 0.020 *	R = 0.33; *p* = 0.07

* Statistically significant correlations. CRP, C-reactive protein; KIM-1, kidney injury molecule-1; L-FABP, liver-type fatty acid-binding protein; uPAR, urokinase-type plasminogen activator receptor.
